# Bone health in childhood and adolescence: an overview on dual-energy X-ray absorptiometry scanning, fracture surveillance and bisphosphonate therapy for low-middle-income countries

**DOI:** 10.3389/fendo.2023.1082413

**Published:** 2023-04-17

**Authors:** Dilki Madhuchani, Sumudu Nimali Seneviratne, Leanne M. Ward

**Affiliations:** ^1^ Faculty of Medicine, University of Colombo, Colombo, Sri Lanka; ^2^ Department of Paediatrics, Faculty of Medicine, University of Colombo, Colombo, Sri Lanka; ^3^ Department of Pediatrics, University of Ottawa and Division of Endocrinology, Children's Hospital of Eastern Ontario, Ottawa, ON, Canada; ^4^ The Ottawa Pediatric Bone Health Research Group, Children's Hospital of Eastern Ontario Research Institute, Ottawa, ON, Canada

**Keywords:** dual energy x-ray absorptiometry, fractures, bone mineral density, paediatric osteoporosis, bone fragility, low-middle-income-countries, corticosteroids, low resource

## Abstract

Bone accrual in childhood determines bone health in later life. Loss of bone strength in early life can lead to increased morbidity and reduced quality of life in childhood and adolescence. Increased availability of assessment tools and bisphosphonate therapy, together with increased awareness on the significance of fracture history and risk factors, have led to greater opportunities, to improve detection and optimize management of children and adolescents with bone fragility globally, including those in lower resource settings. Bone mineral density z-scores and bone mineral content are surrogate measures of bone strength, which can be measured by dual-energy X-ray absorptiometry (DXA), in growing individuals. DXA can aid in the diagnosis and management of primary and secondary bone fragility disorders in childhood. DXA helps evaluate children with clinically significant fractures, and monitor those with bone fragility disorders, or at high risk for compromised bone strength. Obtaining DXA images can however be challenging, especially in younger children, due to difficulty in positioning and movement artefacts, while paediatric DXA interpretation can be confounded by effects of growth and puberty. Furthermore, access to DXA facilities as well as appropriate paediatric reference norms and expertise for interpretation, may not be easily available especially in lower resource settings. Pediatric bone experts are now placing increasing emphasis on the fracture phenotype and clinical context to diagnose osteoporosis over bone mineral density (BMD) by DXA. Low trauma vertebral fractures are now recognized as a hallmark of bone fragility, and spinal fracture surveillance by either conventional lateral thoracolumbar radiographs or vertebral fracture assessment by DXA is gaining increasing importance in diagnosing childhood osteoporosis, and initiating bone protective therapy. Furthermore, it is now understood that even a single, low-trauma long bone fracture can signal osteoporosis in those with risk factors for bone fragility. Intravenous bisphosphonate therapy is the mainstay of treatment for childhood bone fragility disorders. Other supportive measures to improve bone strength include optimizing nutrition, encouraging weight bearing physical activity within the limits of the underlying condition, and treating any associated endocrinopathies. With this paradigm shift in childhood osteoporosis evaluation and management, lack of DXA facilities to assess BMD at baseline and/or provide serial monitoring is not a major barrier for initiating IV bisphosphonate therapy in children in whom it is clinically indicated and would benefit from its use. DXA is useful, however, to monitor treatment response and optimal timing for treatment discontinuation in children with transient risk factors for osteoporosis. Overall, there is lack of awareness and paucity of guidelines on utilizing and adopting available resources to manage paediatric bone disorders optimally in lower-resource settings. We provide an evidence-based approach to the assessment and management of bone fragility disorders in children and adolescents, with appropriate considerations for lower resource settings including LMIC countries.

## 1 Background

Bone is a dynamic tissue in the body, which serves many functions including providing structure, anchoring muscles, protecting internal organs, and storing calcium. Childhood and adolescence are critical periods for bone growth and bone mineral accrual. Bone mineralization starts from antenatal life, and continues until late in the third decade, when peak bone mass is achieved (1, 2). Genetic factors, gender, nutrition, hormones, physical activity, medical conditions, and use of certain medication can all influence bone growth (3). Sub-optimal bone growth in children can lead to painful and debilitating fractures during childhood as well as adulthood (1).

Dual-energy X-ray absorptiometry (DXA) is the most popular imaging modality used to assess bone mineral density (BMD) and fracture risk. It was initially developed using a single beam of X-ray by John Cameron and James Sorenson in 1963 (4). Until the mid-1980s, bone assessment using DXA scan was used only for research purposes, but came in to clinical practice thereafter (5). The DXA machine used in present day practice emits two X-ray beams, one of high-energy, and the other of low-energy. The radiation dose involved in obtaining a DXA scan is between 0.1-6 micro-Sieverts which is equivalent to 1-10% radiation dose of a typical chest X-ray (6). This amount of radiation is equal to background radiation exposure during a single day at sea level (2). Thus, DXA is considered a safe low radiation imaging modality for children, including infants and toddlers (7, 8).

Bisphosphonates (BP) are a class of drugs which facilitate bone mineral accrual by inhibiting osteoclast function in bone (9, 10). They have been used to treat conditions associated with increased bone fragility since the 1960s (10). Bisphosphonates came into paediatric practice in the 1990s following the effective use of intravenous pamidronate to treat children with osteogenesis imperfecta (OI) (11). Current recommendations for use of BP in children with different conditions associated with impaired bone health are discussed below.

## 2 Utilizing DXA to assess bone health in children and adolescents

Bone Mineral Content (BMC) and areal Bone Mineral Density (aBMD) are two important DXA measures which are used for assessing bone health in children and adolescents. aBMD is a DXA derived measure calculated by dividing BMC (in grams) by projected bone surface area (in cm^2^). Both BMC and aBMD are useful in predicting fracture risk (12, 13). However, aBMD is used more often by clinicians, because of easy availability of reference values across all age groups for children (6). For evaluating BMD in children and adolescents, the ISCD 2019 official position recommends the DXA derived measure of Total Body Less Head (TBLH) and the anterior posterior spine (14). Other sites such as the proximal femur, 1/3^rd^ radius and lateral distal femur can be used to measure BMD if assessing standard sites are not feasible, and appropriate reference DXA data are available (14).

In children and adolescents, the BMD Z-score is used for interpreting DXA results. The Z-score indicates the amount of deviation of the measured BMD, from mean BMD of the gender- and age-matched population (2). When the Z-score is ≤ -2, it is interpreted as ‘low bone mass’ or ‘low BMD for age’. The T-score which is reported in DXA reports in adults, and used to defining “osteoporosis” in adults, is not relevant for paediatric practice, as the T-score compares against peak bone mass, and children are yet to gain peak bone mass (2). In children with short stature or delayed puberty, the actual bone size can be smaller than that predicted by the DXA machine based on chronological age, and thus the BMD Z-score can be falsely low. This can be overcome by using z-score for height or stage of puberty, rather than chronological age (2, 6, 15). Ethnic variation in bone accrual have been reported by several multiethnic studies assessing densitometry in children (16, 17). Sex- and ethnic-specific (White, South Asian, Black Afro-Caribbean) reference curves for age and size adjusted lumbar spine and total body densitometry up to the age of 20 years are now available for Hologic and GE Lunar scanners (16). More recently DXA scanners have also been utilized for vertebral fracture assessment using high resolution lateral spine images (18).

### 2.1 Indications for DXA in children

When deciding to perform a DXA scan, several factors including age, family history, fracture history, chronic medical conditions, medication history, and whether the DXA results would influence management, need to be considered (6). Indications for DXA include: 1) assessment of a child with a significant fracture history; 2) diagnosis/monitoring of impaired bone health in children with chronic disease/medication known to increase bone fragility and 3) monitoring of BMD while on bisphosphonate therapy (2).

#### 2.1.1 Clinically significant fracture history in childhood/adolescence

In an apparently healthy child (without other evidence of a primary/secondary bone disorder) with a single fracture or history of recurrent fractures, a comprehensive clinical history focusing on mechanism of injury is important to first determine whether they are pathological fracture/s, attributable to increased bone fragility. Fractures obtained during: road traffic accidents; fall from a height greater than 3m, blunt trauma, and sports-related injuries are considered as high-energy trauma (non-pathological) fractures, and do not generally warrant further evaluation for bone fragility. Thus, otherwise healthy children/adolescents should undergo DXA assessment only if they are suspected to have a pathological fracture due to increased bone fragility, and would benefit from intervention (6).

#### 2.1.2 Evaluation of impaired bone health in children with chronic diseases/medication

Children and adolescents with certain chronic diseases can have impaired bone health due to multiple reasons. These include systemic inflammation, poor nutrition, restricted physical activity, lack of sun exposure, GC exposure, leukemia and other malignancies, and hormone imbalance (19). Clinicians need to be aware about bone health issues, and identify children who are at risk early, to prevent sequelae.

While DXA is a useful tool to identify children at risk of secondary bone fragility due to long standing illness, there are certain limitations to consider. These include: the need to factor in the impact of impaired growth and delayed puberty due to effects of chronic disease, malnutrition and medications during interpretation, and difficulties in positioning and obtaining standard DXA images in children with contractures, indwelling instruments like nasogastric tubes and bone prosthesis (6). Optimal time for DXA monitoring assessments are also uncertain and recommendations vary from disease to disease (20).

##### 2.1.2.1 Glucocorticoid therapy

Short-and long-term glucocorticoids (GCs) are used in the treatment of many childhood diseases. Cumulative dose of systemic GCs, and treatment duration are the two main determinants of impact on bone strength (20). It is well known that the high dose GCs predispose to increased bone fragility. Low to medium doses can also cause detrimental effects on bone, but evidence in children is inconclusive (15, 20). GC treatment predispose to vertebral fractures because GCs affect trabecular bone (main part of spine) more than cortical bone (21). Even GC treated-children with normal DXA results can suffer vertebral fractures (19). Vertebral fractures are frequently asymptomatic particularly in the earlier phase, although they can present with back pain (21).Therefore, in addition to DXA, plain lateral thoracic spine and lumbar spine radiographs are recommended at the start of GC treatment and repeated annually while GC therapy continues (20, 21).

##### 2.1.2.2 Chronic inflammatory disorders

Children with chronic inflammatory conditions such as inflammatory bowel disease (IBD) and juvenile idiopathic arthritis (JIA), can have impaired bone health due to chronic inflammatory mediators (6, 19). Prevalence of osteoporosis among children with IBD is reported to be 40% higher compared to age- and sex-matched healthy population (22). Likewise, increased bone fragility is observed among children and adolescents across all subtypes of JIA (23). Therefore, secondary osteoporosis is a major problem in childhood IBD and JIA, and guidelines for monitoring bone health in children with these conditions are now available (20, 22).

##### 2.1.2.3 Malignancy

Children with cancer are well recognized to be at risk of bone fragility due to the disease as well as its treatment. Potential factors which contribute to bone fragility include osteotoxic substances produced by cancer cells, chemotherapy with osteotoxic drugs (methotrexate, cyclosporin, glucocorticoids etc.), total body irradiation, cranial irradiation causing pituitary hormone deficiencies (eg: hypogonadism, growth hormone deficiency), malnutrition and immobility (6, 19, 24). However, bone health of these children is often neglected due to the severity and complexity of their underlying condition which is given priority in treatment paradigms.

Children with acute lymphoblastic leukemia (ALL), particularly GC-treated children have a relatively high incidence of vertebral fractures (19). In childhood ALL, 16% are reported to have a symptomatic/asymptomatic vertebral fracture at the time of diagnosis, and the incidence increases further, especially during periods of high glucocorticoid exposure (15, 19, 25). Presence of a vertebral fracture/s at the time of diagnosis also indicates higher risk of acquiring new vertebral as well as non-vertebral fractures. Nevertheless, most children with leukemia appear to have the potential to undergo vertebral body reshaping to reclaim normal vertebral dimensions over time, except for older adolescents (with less residual growth potential) and those with more severe vertebral collapse (12). The International Late Effects of Childhood Cancer Guideline Harmonization Group (IGHG) in 2021 recommends baseline DXA between 2-5 years after completing cancer treatment, and again at 25 years of age when bone mass peaks, or earlier depending on risk factors (24).

##### 2.1.2.4 Chronic immobilization

Chronic immobilization is another factor which impairs bone strength, where reduced mechanical loading on the muscle-bone unit reduces muscle strength as well as bone strength (15). Therefore, children with cerebral palsy (CP), congenital myopathies such as Duchenne muscular dystrophy (DMD) and other conditions associated with impaired mobility are predisposed to fragility fractures (6, 19, 21). In children with contractures, spinal deformities, and bone prosthesis, positioning and interpretation of DXA/radiographs can be difficult. In these situations, BMD assessment using the distal femur is a suitable alternative (6, 19, 20).

Among children with CP, poor nutrition due to feeding difficulties, and anti-epileptic drugs can also contribute to reduced BMD in addition to reduced mobility (19). Vertebral fractures are common in children with CP, as well as distal femur fractures (21). Children with DMD often lose their mobility when they reach adolescence (19). Treatment of children with DMD with GCs also increases their risk of fractures. Vertebral fractures can occur even with normal BMD, particularly in GC treated children (26). Therefore, vertebral fracture assessment using spine radiographs are recommended at baseline (6 to 8 years of age) and then 1- 2 yearly thereafter if on GCs, or 2 -3 yearly if not on GCs, as well as if they develop back pain (20, 21).

##### 2.1.2.5 Thalassemia

In thalassemia, bone marrow expansion to compensate for ineffective erythropoiesis results in reduced trabecular bone and cortical thinning which leads to increased fragility of bone (27). In addition, iron overload and deposition in the pituitary gland can cause pituitary hormone deficiencies and growth failure (27). Thus, children with thalassemia are at increased risk of fragility fractures, and DXA assessments are recommended from adolescence (20).

#### 2.1.3 Therapeutic monitoring of children while on bisphosphonates

DXA is used for monitoring BMD as a measure of efficacy of bisphosphonate treatment, with baseline DXA at the start of treatment and repeat scans at 6-12 monthly intervals (6, 20). However, the relationship between change in BMD, and reduction in fracture risk is yet unclear. A reasonable goal is to achieve an aBMD Z-score (if previously low) that approximates the patient’s height Z-score, given the size-dependent nature of areal BMD measurements.

## 3 Childhood osteoporosis

The diagnosis of childhood osteoporosis has recently moved from a DXA-based approach employed in adult practice, to a fracture based approach. This paradigm shift occurred mainly due to issues arising based on variability in BMD Z scores when using different normative datasets, as well as observation of occurrence of low trauma vertebral fractures even with BMD Z scores > -2 (21). “Childhood osteoporosis’ is thus diagnosed only in children with pathological fractures now (15). In children with known risk factors for either primary osteoporosis: such as evidence of a primary genetic bone fragility disorder such as OI (positive family history, blue sclera, limb deformities, dentigenosis imperfecta); or secondary osteoporosis (long term high dose glucocorticoids, chronic neuromuscular disorders ect.), the presence of a single low-trauma fracture warrants a diagnosis of osteoporosis (25, 26, 28–30). In children with secondary osteoporosis, low trauma is defined as falling from a standing height or less, at no more than walking speed.

In otherwise *healthy* children and adolescents (without apparent evidence of a primary genetic bone fragility disorder or chronic disease or medication associated with bone fragility) ‘osteoporosis’ is diagnosed by the presence of either: (A) a low trauma vertebral fracture (irrespective of the DXA results); or (B) low BMD (gender- and age-matched Z-score ≤ -2) with a clinically significant fracture history, defined by (i) ≥ 2 long bone fractures, by 10 years of age, or (ii)≥ 3 long bone fractures, up to 19 years of age (25). 

## 4 Fracture detection/surveillance

Presence of either vertebral fractures or low trauma long bone fractures are now considered an integral part in the diagnosis of childhood osteoporosis. This paradigm change emphasizes the importance of fracture surveillance in bone health monitoring in children. Vertebral fractures are considered the hallmark of bone fragility in children and adolescents. Spinal imaging by lateral thoracolumbar spine radiographs, or “vertebral fracture assessment” (VFA) by DXA is now becoming an integral part of osteoporosis assessment.

**Figure 1:**
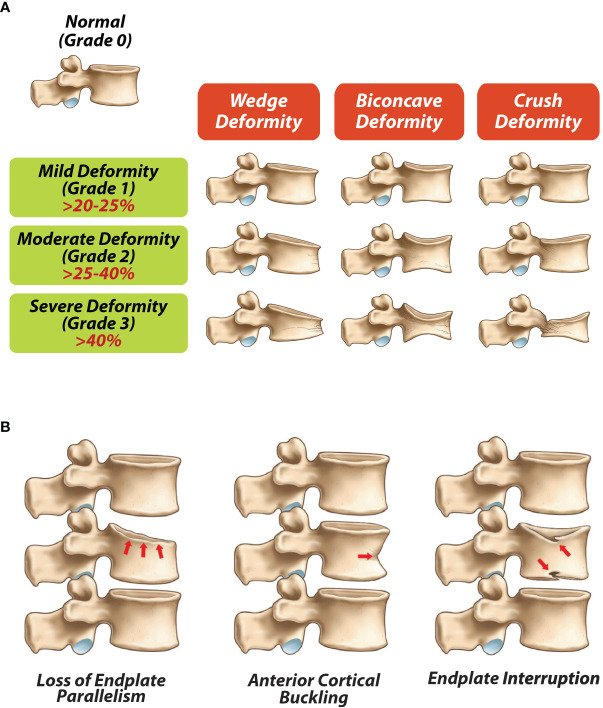
**(A)** Modified Genant semi-quantitave classification for vertebral frature assessments (adapted from Genant et al (31), **(B)** Qualitative radiological signs of vertebral fractures (adapted from Ward LM (25).

Vertebral fracture evaluations by conventional radiography can be applied reliably using a semi quantitative approach as described by Genant et al. with loss of >20% of vertebral height denoting a vertebral facture in children (**Figure 1A**). Using this method, vertebral fractures are classified based on severity as grade 1/mild (> 20 –25% loss of vertebral height ratio), grade 2/moderate (> 25–40% loss of vertebral height ratio), and grade 3/severe (> 40% loss of vertebral height ratio). This method has been validated for use in children, including the fact that Genant-defined fractures predict future vertebral and long bone fractures in children with chronic GC-treated disorders (30). Additionally, qualitative signs such as loss of end plate parallelism, anterior cortical buckling and end plate interruption (**Figure 1B**) can be used to differentiate fractures from physiological rounding where necessary (25, 31, 32). Modern DXA scanners are technically capable of producing high resolution lateral spine images for VFA. DXA-based VFA can be used instead of spine radiography to identify symptomatic and asymptomatic vertebral fractures, provided the evaluator has experience in the assessment of pediatric VF and the images are of high enough quality to adequately determine vertebral body morphology (18). Advantages of DXA VFA over conventional radiography include greater patient convenience and less radiation exposure (33).

Low trauma vertebral fractures are particularly common among children with secondary osteoporosis including those on GC therapy, neuromuscular and inflammatory disorders and leukemia, and are frequently asymptomatic (25). With respect to long bone fractures, those involving the lower extremities generally cause the most significant morbidity due to effects on mobility, transfer and self-care. Low-trauma fractures involving the femur and humerus are typically a clear indicator of bone fragility, while single fractures involving the tibia as well as comminuted and atypically displaced fractures, are also frequently significant. Forearm fractures (which are common in heathy children following trauma) and those involving the fingers and toes should be considered on the background of associated risk factors, and may carry less weight in deciding whether to commence BP therapy (25) depending on the degree of trauma and the likelihood of true bone fragility given the overall clinical context. Useful algorithms to aid monitoring of children with chronic disease are now available (25).

## 5 Bisphosphate therapy in childhood and adolescence

Bisphosphonates (BPs) are synthetic derivatives of pyrophosphates. They increase bone mineral density by inhibiting osteoclast action, and preventing bone resorption. Oral BPs are poorly absorbed from the gastro-intestinal system (34). They are selectively concentrated in high bone remodeling areas with slow elimination from bone, and finally excreted from the body *via* the kidneys (20). BPs now play a well-established and significant role in the management of children with primary osteoporosis (e.g., osteogenesis imperfecta) and more recently, in those with secondary osteoporosis when spontaneous resolution appears unlikely.

Typically, a secondary prevention approach is employed, whereby BP therapy is usually commenced following a fragility fracture in children/adolescents who are unlikely to recover spontaneously from osteoporosis without medication (20). In these patients, even a single low trauma fracture warrants consideration of pharmacological therapy. There is no clear evidence for BP use in primary prevention of fragility fractures, although some experts recommend starting BP in high-risk children who do not have overt bone fragility, but have “low BMD in early puberty, with low Z-scores and decreasing trajectories” (20, 21). Notably, it is also very important to institute other measures to optimize bone health as necessary. These can include optimizing nutrition, especially factors which are important for bone health (e.g., vitamin D, calcium), encouraging weight bearing physical activity, primary prevention of trauma/falls in vulnerable children, minimizing use of osteotoxic drugs, correcting hormone deficiencies, and optimizing disease control of the primary disease in secondary bone fragility disorders (20, 21).

Many studies have shown that intravenous (IV) BPs (e.g. pamidronate, zoledronic acid) are more efficacious in children than oral BPs (e.g. alendronate, risedronate) (1, 20, 26, 35). IV zoledronic acid is 100 times more potent than IV pamidronate, and requires lower dose as well as less frequent dosing (21). Recommended maximum annual dose of: IV pamidronate is 9 mg/kg; and IV zoledronic acid is 0.1 mg/kg (9, 21, 36). Bisphosphonate therapy has short term and long-term side effects. An “acute phase reaction” following initial doses of BP is commonly observed with clinical features such as fever, malaise, back pain, body pains, nausea, and vomiting. Asymptomatic hypocalcemia is another short-term side effect (21). There is less robust data on long term side effects at present.

Due to side effects experienced more commonly with first ever dose of BP, a starting dose of 0.5 mg/kg for IV pamidronate and 0.0125 mg/kg or 0.025 mg/kg for IV zoledronic acid is recommended (9). In younger children, due to high bone turnover, more frequent dosing of BPs is practiced (IV pamidronate: 2-monthly in children aged <2 years, 3-monthly in children aged 2-3 years and 4-monthly in children > 3 years, IV zolendronic acid as 4 divided doses, every 3-monthly in children below 2 years) (21). In older children, the annual IV pamidronate dose can be given in 4-6 divided doses, while IV zoledronic acid can be given in 2 divided doses per year, at the start of treatment (9).

In children with genetic bone fragility, early start of BPs (within first 2 to 3 weeks of life) in neonates presenting with in-utero fractures and deformities, is practiced in some centers, and has shown favorable outcomes (36, 37). In children with secondary osteoporosis, the likelihood of spontaneous recovery from osteoporosis due to resolution of risk factors should also be considered, before commencing BP therapy. These include better control/resolution of the underlying primary disease condition/cessation of GC therapy. Duration of therapy will also depend upon continuation/resolution of the underlying risk factor/s leading to secondary osteoporosis.

In the absence of robust research data to guide the optimal duration of therapy, the current approach is to treat with high doses until the patient reaches ‘clinical stability’’; the typical duration for this phase of treatment is often two years (the stabilization phase). Once osteoporosis has stabilized (defined as absence of new or worsening of vertebral fractures, absence of new long bone fractures plus improvements in BMD trajectories and mobility), the patient may be transitioned to the maintenance phase, with the goal of retaining the benefits achieved during the stabilization phase (often at a lower dose than in the stabilization phase), until risk factors resolve or linear growth ceases. In this phase, the BMD Z-score trajectory is useful for informing dose titrations (with the goal to achieve a normal rate of bone and mineral accrual) (9, 21). Downward dose titration can be done either by reducing the dose, or increasing the interval between doses. In those with secondary osteoporosis discontinuation of therapy can be considered when the patient has been fracture free for at least 6 -12 months following resolution of risk factors, provided the bone mineral accrual Z-score trajectory has also normalized.

Monitoring the efficacy of bisphosphonate therapy should be based on functional clinical parameters as well as DXA measures (where available). One of the main goals of therapy is remittance of back and bone pain which typically occurs within 2 to 6 weeks following the first dose, but which may recur in the days to weeks leading up to subsequent doses. Other features indicating ‘clinical stability’ include absence of new vertebral fractures/further loss of vertebral height at sites of previous fractures, reshaping of vertebral fractures, absence of new non-vertebral fractures, improved mobility and increase in spine BMD Z-score appropriate for height (21).

## 6 Adaptations for low-middle-income countries

**Figure 2:**
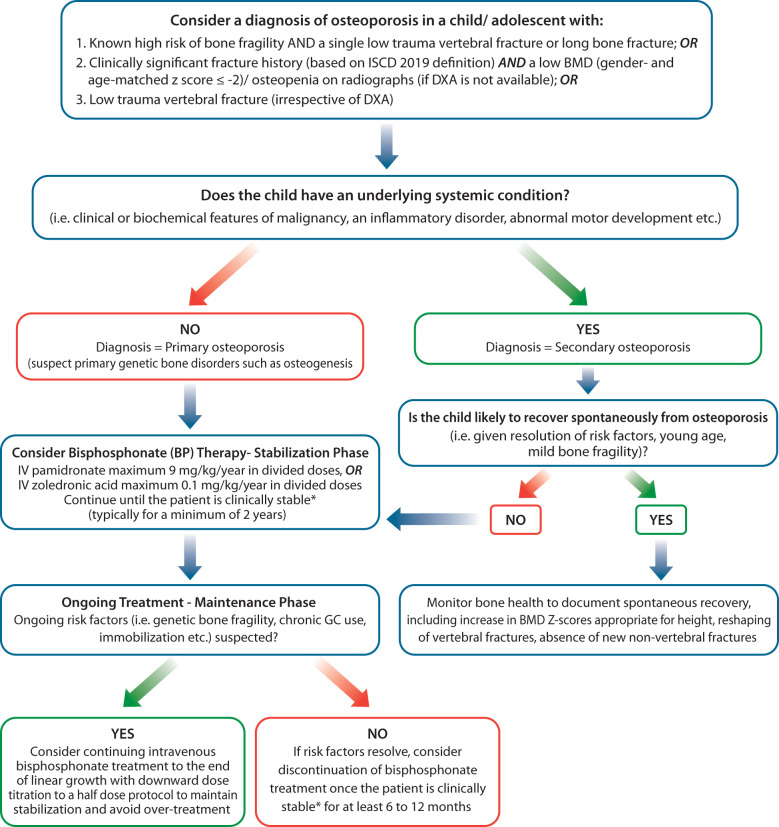
Algorithm for evaluation and management of bone fragility in children in Low-middle-income-countries. *Clinically stable includes: 1) Absence of new VF in previously normal vertebral bodies and absence of further loss of vertebral height at sites of previous fractures, 2) Reshaping of vertebral fractures, 3) Absence of new non-vertebral fractures, bone pain and back pain, 4) Improved mobility, increases in spine BMD Z-score appropriate for height.

As discussed above, in recent years, the diagnosis, monitoring and treatment of pediatric osteoporosis has moved away from a DXA-focused to a fracture-focused approach (28). This shift in fact, is favorable to LMIC, due to several reasons. While facilities for childhood DXA scanning and interpretation are still unlikely to be easily available in many LMICs yet, plain radiography for assessing fractures tends to be accessible around the world. Further, there is lack of robust evidence to ascertain if whether paediatric reference norms derived from children from high income countries are appropriate for children from LMICs. Based on the new paradigm, children with known risk factors for osteoporosis, such as family history or other clinical manifestations of genetic bone fragility conditions or chronic illnesses known to be associated with osteoporosis, a single low-trauma vertebral or a single long bone fracture on plain radiography is sufficient to diagnosis osteoporosis (25, 29). The presence of a low trauma vertebral fracture in an otherwise healthy child/adolescent also warrants a diagnosis of osteoporosis. As such, the only condition where DXA is needed for establishment of childhood of osteoporosis is in otherwise healthy children who lack evidence of a primary or secondary bone fragility disorder, but have suffered two or more long bone fractures by age 10 or three or more long bone fractures by age 19, not attributable to high energy trauma. In such cases, the ISCD recommends DXA scanning to ascertain that the child has a low BMD (gender- and age-matched Z-score ≤ -2) to prevent over-diagnosis of osteoporosis in children who have simply been unlucky during play or sports. If DXA facilities are not available to affirm a bone fragility condition in otherwise healthy children with fractures, plain radiography films can be used to evaluate for the PRESENCE of osteopenia as well as disorders of EXCESS bone mass (suggestive of osteoPETROSIS), since bone fragility can occur at both ends of the BMD spectrum (See **Figure 2**).

The relationship between change in BMD and reduction in fracture risk is yet unclear, and inability to perform serial DXA monitoring should not be a barrier to BP therapy. Monitoring fracture rates are important in all patients, and can be utilized instead of serial DXA measures if access to DXA scanning is limited. In the absence of an available DXA scanner, it is reasonable to treat a patient at published starting doses until the osteoporosis stabilizes (see **Figure 2**), and then down-titrate IV bisphosphonate dosing until risk factors resolve (at which time, bisphosphonate therapy can be discontinued). If risk factors do not resolve, bisphosphonate therapy can be continued at a down-titrated dose to achieve treatment goals (improved pain, and mobility, reduction in new non-vertebral and vertebral fracture rates, reshaping of previously fractured vertebral bodies). As such, plain radiography can be utilized for the diagnostic and treatment monitoring by centers which lack access to DXA scanning facilities.

There is robust evidence to support the use of intravenous versus over oral bisphosphate therapy in the management of childhood osteoporosis (21). When considering which BP agents are more appropriate for the management of childhood osteoporosis in LMICs, intravenous BP agents including pamidronate and zolendronic acid, although more expensive than oral BP therapy, are more potent and have demonstrated a more favorable impact on BMD in children compared with oral BP therapy (21). However, in the absence of available intravenous therapy, oral therapy such as alendronate could be considered where appropriate, although there is less evidence of efficacy. There could be a place for oral BPs such as alendronate in children with OI with mild disease or in children with stable disease for some time (9, 20, 36).

DXA is useful for monitoring BMD in children on bisphosphonates. If facilities for BMD monitoring are not available, children on therapy will need to be carefully monitored for clinical stability, using clinical and plain radiographic surveillance for (1) absence of back and bone pain (2) lack of incident vertebral and long bone fractures, and (3) reshaping of vertebral bodies where there is sufficient growth to support this physiological process. In such cases, it is paramount to avoid excessive bisphosphonate doses, as the absence of DXA will preclude the availability to avoid the high bone mass that can come with over-treatment. Two years of stabilization with pamidronate (9 mg/kg/year) or zoledronic acid (0.1 mg/kg/year) followed by downward dose titration (for example, to a half-dose protocol), or starting with a half-dose protocol and down-titrating further, will help ensure lack of over-treatment in the absence of corroborative DXA monitoring (**Figure 2**).

## 7 Conclusion

Greater opportunities and options for evaluation and management for children and adolescents with impaired bone health are now increasing available globally. There are substantial differences in evaluation and management of impaired bone health in children and adolescents, in contrast to adults. These include differences in defining “osteoporosis”, greater complexity in DXA interpretation due to continuous changes brought about by growth and puberty, and focus on commencing IV BP as first-line of therapy following clinically significant fracture/s in a child/adolescent who is unlikely to have spontaneous resolution. We propose that increased awareness and simplified management guidelines for low-resource settings, as provided in this article, can help improve bone health in children and adolescents with primary and secondary bone fragility disorders living in LMICs.

## Author contributions

DM reviewed the literature and wrote the initial manuscript. SS conceptualized the project, provided guidance on writing and revising the initial manuscript, and did subsequent revisions. LW helped draft and revise the manuscript and provided guidance and mentorship at all stages. All authors contributed to the article and approved the submitted version of the manuscript.

## Funding

LMW is supported by a Tier 1 Clinical Research Chair in Pediatric Bone Disorders from the University of Ottawa and the Children's Hospital of Eastern Ontario Research Institute.
